# Prevalence of extended-spectrum cephalosporin-resistant *Escherichia coli* in a farrowing farm: ST1121 clone harboring IncHI2 plasmid contributes to the dissemination of *bla*_CMY-2_

**DOI:** 10.3389/fmicb.2015.01210

**Published:** 2015-11-03

**Authors:** Hui Deng, Hong-Bin Si, Shu-Yi Zeng, Jian Sun, Liang-Xing Fang, Run-Shi Yang, Ya-Hong Liu, Xiao-Ping Liao

**Affiliations:** ^1^National Risk Assessment Laboratory for Antimicrobial Resistance of Animal Original Bacteria, South China Agricultural UniversityGuangzhou, China; ^2^College of Veterinary Medicine, South China Agricultural UniversityGuangzhou, China; ^3^College of Animal Science and Technology, Guangxi UniversityNanning, China; ^4^Jiangsu Co-Innovation Centre for Prevention and Control of Important Animal Infectious Diseases and ZoonosesYangzhou, China

**Keywords:** *Escherichia coli*, clonal spread, *bla*_CMY-2_, IncHI2 plasmid, farrowing farm

## Abstract

During a regular monitoring of antimicrobial resistance in a farrowing farm in Southern China, 117 *Escherichia coli* isolates were obtained from sows and piglets. Compared with the isolates from piglets, the isolates from sows exhibited higher resistance rates to the tested cephalosporins. Correspondingly, the total detection rate of the *bla*_CMY-2_/*bla*_CTX-M_ genes in the sow isolates (34.2%) was also significantly higher than that of the piglet isolates (13.6%; *p* < 0.05). The *bla*_CMY-2_ gene had a relatively high prevalence (11.1%) in the *E. coli* isolates. MLST and PFGE analysis revealed the clonal spread of ST1121 *E. coli* in most (7/13) of the *bla*_CMY-2_-positive isolates. An indistinguishable IncHI2 plasmid harboring *bla*_CMY-2_ was also identified in each of the seven ST1121 *E. coli* isolates. Complete sequence analysis of this IncHI2 plasmid (pEC5207) revealed that pEC5207 may have originated through recombination of an IncHI2 plasmid with a *bla*_CMY-2_-carrying IncA/C plasmid like pCFSAN007427_01. In addition to *bla*_CMY-2_, pEC5207 also carried other resistance determinants for aminoglycosides (*aacA7*), sulfonamides (*sul1*), as well as heavy metals ions, such as Cu and Ag. The susceptibility testing showed that the pEC5207 can mediate both antibiotic and heavy metal resistance. This highlights the role of pEC5207 in co-selection of *bla*_CMY-2_-positive isolates under the selective pressure of heavy metals, cephalosporins, and other antimicrobials. In conclusion, clonal spread of an ST1121 type *E. coli* strain harboring an IncHI2 plasmid contributed to the dissemination of *bla*_CMY-2_ in a farrowing farm in Southern China. We also have determined the first complete sequence analysis of a *bla*_CMY-2_-carrying IncHI2 plasmid.

## Introduction

Antimicrobial agents are often used as feed and water additives in food animals to treat or prevent disease and to promote general overall health (McEwen and Fedorka-Cray, 2002; Cabello et al., 2013). However, antimicrobial resistance driven by the intensive use of antimicrobial agents in animal husbandry is increasing worldwide (Witte, 1998). In the swine industry, antimicrobial resistance patterns can be traced to particular farms associated with certain management practices (Rasschaert et al., 2012; Brooks et al., 2014). In farrowing farms, antimicrobial resistance in piglets has been shown to be a reflection of antimicrobial use in sows (Mathew et al., 2005; Callens et al., 2015), because sows are an important reservoir of antimicrobial resistant bacteria for their offspring (Callens et al., 2012; Crombe et al., 2013). Additionally, piglet transfer from farrowing to finishing farms increases the likelihood of the transmission of resistant bacteria, thus increasing the risk of antimicrobial resistance transfer between swine farms (Sandvang et al., 2000; van Duijkeren et al., 2008). Therefore, surveillance for antimicrobial resistance in the farrowing farm is important for controlling the dissemination of antimicrobial resistance.

*Escherichia coli* is an important cause of intestinal and extraintestinal diseases in animals and humans worldwide, and β-lactams are widely used in veterinary medicine to treat colibacillosis. However, the use of extended-spectrum cephalosporins (ESCs) in animals has contributed to β-lactam resistance in *E. coli* (Greko et al., 2009). Resistance to ESCs in *E. coli* has been associated with the extended-spectrum β-lactamases (ESBLs) and plasmid-mediated Ambler class C cephamycinases (pAmpC β-lactamases; Seiffert et al., 2013). ESBLs are the major contributors to ESC resistance in *E. coli* and confer resistance to cephalosporins with an oxyimino side chain (cefotaxime, ceftriaxone, and ceftazidime; Bradford, 2001).

Unlike ESBLs, pAmpC β-lactamases exhibit activity against cephamycins, such as cefoxitin and cannot be inactivated by β-lactamase inhibitor clavulanate (Bradford, 2001; Jacoby, 2009). Among them, CMY-2, the most common pAmpC β-lactamase, has been documented worldwide in bacteria of human and animal origin (Li et al., 2007; Jacoby, 2009). The *bla*_CMY-2_ gene which likely originated from the chromosomal AmpC locus of *Citrobacter freundii* has been horizontally transmitted through plasmids in *E. coli* from different sources (Martin et al., 2012). Various plasmid types are associated with *bla*_CMY-2_, including IncA/C, IncF, IncI1, IncL/M, IncP, IncK, and IncHI2. Of these, IncA/C and IncI1 plasmid are the most common carriers of *bla*_CMY-2_ (Verdet et al., 2009; Folster et al., 2011; Martin et al., 2012; Bortolaia et al., 2014; Guo et al., 2014).

The *bla*_CMY-2_ gene is prevalent in ESC-resistant *E. coli* of livestock (Li et al., 2007; Seiffert et al., 2013). In swine, the detection rate of *bla*_CMY-2_ in *E. coli* is quite different, ranging from 0 to up to 80% (Seiffert et al., 2013). Although the detection methods of CMY-2 vary between reports, this may still reflect the different prevalence of *bla*_CMY-2_ worldwide. In mainland China, the *bla*_CMY-2_ gene was detected for the first time in *E. coli* of swine origin in Tian et al. (2012), Zheng et al. (2012), and subsequently occured in swine *E. coli* isolates carrying plasmid-mediated quinolone resistance (PMQR) genes (Liu et al., 2013a). Recently, a surveillance study identified *bla*_CMY-2_ mainly in *E. coli* of pig origin, highlighting the role of CMY-2 in the ESC resistance of swine *E. coli* (Guo et al., 2014). In the present study, we make an investigation on the prevalence of drug resistance and ESBL/pAmpC genes in *E. coli* isolates from a swine farm in Southern China. The *bla*_CMY-2_-positive isolates were further analyzed to characterize the transmission mechanisms of the *bla*_CMY-2_ gene.

## Materials and Methods

### Bacterial Isolates and Antimicrobial Susceptibility Testing

In August 2011, a regular monitoring of antimicrobial resistance was conducted in a farrowing farm in Southern China. This farm had been in operation about 8 years and consisted of 2,300 sows with production of about 40,000 piglets for market annually. Rectal swab samples were randomly taken from one pig in every batch pen. The rectal swabs were taken by inserting the sterile swab about 2 cm into the rectum, rotated gently and then immersed in sterile PBS. After collection, the swabs were immediately brought to the laboratory in cool conditions. A total of 137 swab samples were collected from sows (1–5 years-old) and piglets (1–60 days-old). The collected samples were plated on MacConkey agar and then incubated at 37°C for 24 h. One suspected colony with typical *E. coli* morphology was selected from each sample and was identified with API 20E system (BioMerieux, France). Minimal inhibitory concentrations (MICs) of ampicillin (AMP), ceftazidime (CAZ), cefoxitin (FOX), cefotaxime (CTX), ceftiofur (CEF), amikacin (AMK), kanamycin (KAN), florfenicol (FFC), doxycycline (DOX), enrofloxacin (ENR), and trimethoprim-sulfamethoxazole (SXT) were determined by the agar dilution method in accordance with the standard provided by the Clinical and Laboratory Standards Institute (2013a,b). *E. coli* ATCC 25922 was used as the quality control strain.

### Detection of ESBL/pAmpC Genes

Extended-spectrum β-lactamase genes (*bla*_TEM_, *bla*_SHV_, *bla*_CTX-M-1G_, *bla*_CTX-M-9G_, *bla*_CTX-M-2G_, and *bla*_CTX-M-25G_) among the *E. coli* isolates were analyzed by PCR amplification using previously published primers and protocols (Liu et al., 2013a). Purified PCR products were sequenced and compared using the β-lactamase classification system^1^ to confirm the subtypes. Detection of pAmpC genes was performed by a multiplex PCR as previously described (Perez-Perez and Hanson, 2002). For amplification of the entire *bla*_CMY-2_ gene, PCR-positive isolates were re-amplified and sequenced with specific primers (Perez-Perez and Hanson, 2002).

### Molecular Typing

All *bla*_CMY-2_-positive isolates were classified according to *XbaI*-pulsed-field gel electrophoresis (PFGE) type (Tenover et al., 1995). Comparison of PFGE patterns was performed by BioNumerics^®^v6.6 (Applied Maths, Ghent, Belgium) with a cut-off at 90% of the similarity values to indicate identical PFGE types. Multilocus sequence typing (MLST) was performed by using the primers and protocol specified at the *E. coli* MLST web site^2^.

### Transferability of *bla*_CMY-2_

Conjugation experiments were performed as previously described (Chen et al., 2007a), using *E. coli* C600 (streptomycin-resistant; MIC >2000 μg/ml) as a recipient. Putative transconjugants were selected on MacConkey agar plates with streptomycin (2000 μg/ml) and cefoxitin (32 μg/ml), examined for the presence of *bla*_CMY-2_ by PCR assay, and finally confirmed by ERIC-PCR (Versalovic et al., 1991). All transconjugants were tested for antimicrobial susceptibility as described above.

The susceptibility of transconjugant EC5207-35T to heavy metals (Cu, Ag) was tested by microdilution in an aerobic atmosphere, as previously described, with some modifications (Mourao et al., 2015). Briefly, the transconjugant was incubated in Mueller–Hinton broth with serial dilutions of CuSO_4_ (0.25, 0.5, 1, 2, 4, 8, 12, 16, 20, 24, 32, and 36 mM, adjusted to pH 7.2) and AgNO_3_ (0.0125, 0.025, 0.06, 0.08, 0.125, 0.16, 0.25, 0.32, 0.5, 1.0, 1.5, and 3 mM, adjusted to pH 7.4). *E. coli* C600 was used as the reference strain.

### Plasmid Analysis

Plasmids of the transconjugants were typed with PCR-based replicon typing (PBRT; Carattoli et al., 2005). The size of *bla*_CMY-2_-carrying plasmid in the transconjugants was determined using S1 nuclease-digested (TaKaRa Biotechnology, Dalian, China) genomic DNA followed by PFGE and Southern blot hybridization with a *bla*_CMY-2_-specific probe (Barton et al., 1995). Plasmid DNA from the transconjugants was extracted using the QIAGEN Plasmid Midi Kit, and was further analyzed by restriction fragment length polymorphism (RFLP) using *XbaI* (TaKaRa Biotechnology, Dalian, China).

### Complete Sequence of *bla*_CMY-2_-carrying IncHI2 Plasmid pEC5207

In order to further characterize the *bla*_CMY-2_-carrying IncHI2 plasmids of the ST1121 clone in this study, the plasmid pEC5207 from transconjugant EC5207-35T was sequenced using SMRT sequencing approach and assembled by HGAP2.2.0 method (Chin et al., 2013). Open reading frames (ORFs) prediction and annotation were performed with the RAST tools (Aziz et al., 2008). Sequence comparison and map generation were performed using BLAST^3^ and Easyfig version 2.1 (Sullivan et al., 2011).

### Statistical Methods

Statistical significance for the comparison of prevalence data was determined by the χ^2^ test. Differences were considered statistically significant at *p* < 0.05.

### Nucleotide Sequence Accession Number

The complete DNA sequence of plasmid pEC5207 was assigned GenBank accession number KT347600.

## Results

### Antimicrobial Susceptibility

Rectal swab samples were randomly taken from one pig in every batch pen and 117 *E. coli* samples were isolated from 137 sow and piglet rectal swabs. The incidence for each group was approximately 85% (**Table 1**). Antimicrobial susceptibility tests showed that part of the isolates were resistant to CEF (30.8%), CTX (27.4%), FOX (14.5%), and CAZ (11.1%). Except for CAZ, cephalosporin resistance rates for sow isolates were significantly higher than those for isolates from piglets (*p* < 0.05). However, the resistance rate to CAZ for isolates from sows was still three times of that for piglet isolates (**Table 2**). In addition to cephalosporins, different levels of resistance to other classes of antimicrobials, including SXT (100%), DOX (98.3%), FFC (96.6%), AMP (94.0%), KAN (83.8%), ENR (64.1%) and AMK (29.9%), were also observed in the tested isolates (**Table 2**). The vast majority of the isolates (95.7%) exhibited a multidrug resistance (MDR) phenotype, and were resistant to four or more tested antimicrobial agents.

**Table 1:** Information on the samples and *Escherichia coli* isolates in this study.

Sample source	No. of samples	No. (%) of *E. coli* isolates
Sow	84	73 (86.9)
Piglet	53	44 (83.0)
Total	137	117 (85.4)

**Table 2:** Susceptibility of 117 *E. coli* isolates to 11 antimicrobial agents.

Antimicrobial agent^a^	MIC range (μg/ml)	MIC_50_ (μg/ml)	MIC_90_ (μg/ml)	Resistant (%)		
				Sow (*n* = 73)	Piglet (*n* = 44)	Total (*n* = 117)
CAZ	0.06–64	0.25	16	15.1	4.5	11.1
FOX	2–256	4	64	20.5	4.5^∗^	14.5
CEF	<0.125–>256	1	128	38.4	18.2^∗^	30.8
CTX	0.03–>256	0.125	32	35.6	13.6^∗^	27.4
AMP	4–>512	>512	>512	94.5	93.2	94.0
AMK	1–>256	2	>256	17.8	50.0^∗^	29.9
KAN	1–>512	>512	>512	78.1	93.2	83.8
FFC	4–512	256	512	95.9	97.7	96.6
DOX	4–256	64	128	97.3	100	98.3
ENR	0.016–128	4	32	60.3	70.5	64.1
SXT	2/38–>16/304	>16/304	>16/304	100	100	100

*^a^CAZ, ceftazidime; FOX, cefoxitin; CEF, ceftiofur; CTX, cefotaxime; AMP, ampicillin; AMK, amikacin; KAN, kanamycin; FFC, florfenicol; DOX, doxycycline; ENR, enrofloxacin; SXT, trimethoprim-sulfamethoxazole.*

*^∗^*p* < 0.05 for resistance rate of isolates from piglets vs. sows.*

### Detection of ESBL/pAmpC Genes

**FIGURE 1:**
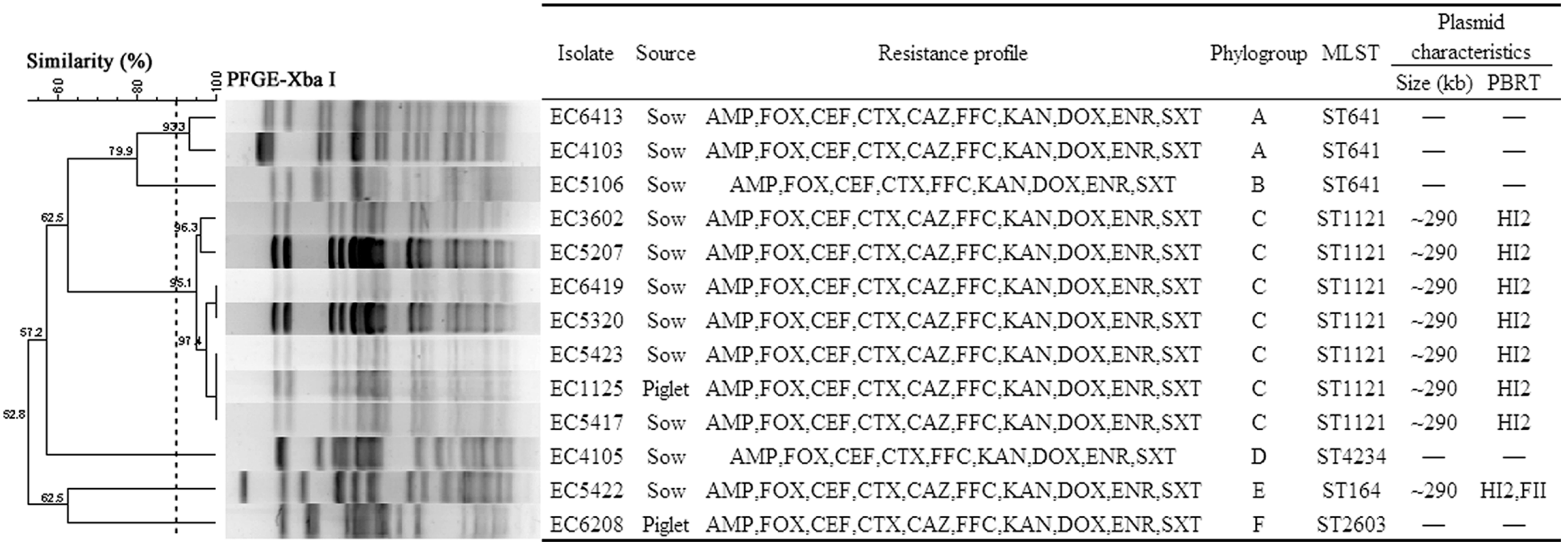
**Clonal relationship and plasmid characteristics of the 13 *bla*_CMY-2_-positive *Escherichia coli* isolates. -, not detected.** AMP, CAZ, FOX, CEF, CTX, KAN, FFC, DOX, ENR, and SXT are represented as in **Table 2**.

Among the 117 *E. coli* isolates, CTX-M-type ESBL genes were detected in 18 isolates (15.4%). The most common *bla*_CTX-M_-type were *bla*_CTX-M-122_ (*n* = 8), followed by *bla*_CTX-M-65_ (*n* = 5), *bla*_CTX-M-55_ (*n* = 3) and *bla*_CTX-M-64_ (*n* = 2). TEM- and SHV-type ESBL genes were not detected in any of the isolates. Compared with the diverse *bla*_CTX-M_ genes detected in this study, *bla*_CMY-2_ was the only identified pAmpC gene, and was found in 13 isolates (11.1%). All the *bla*_CMY-2_-positive isolates were resistant to FOX with MICs ranging from 64 to 256 μg/ml, and also exhibited multi-resistance to AMP, CTX, CEF, KAN, FFC, DOX, ENR, and SXT (**Figure 1**).

The co-existence of ESBL and pAmpC genes was not observed in any of the isolates. The total detection rate of the *bla*_CMY-2_/*bla*_CTX-M_ genes in the sow isolates (34.2%) was significantly higher than that in the piglet isolates (13.6%; *p* < 0.05). The distribution of ESBL/pAmpC genes among the isolates are listed in **Table 3**.

**Table 3:** Distribution of ESBL/pAmpC genes among *E. coli* isolates.

Gene type	No. isolates (%)		Total (*n* = 117)
	Sow (*n* = 73)	Piglet (*n* = 44)	
**ESBL**	14 (19.2)	4 (9.1)	18 (15.4)
*bla*_CTX-M-55_	3 (4.1)		3 (2.6)
*bla*_CTX-M-64_	1 (1.4)	1 (2.3)	2 (1.7)
*bla*_CTX-M-65_	4 (5.5)	1 (2.3)	5 (4.3)
*bla*_CTX-M-122_	6 (8.2)	2 (4.5)	8 (6.8)
**pAmpC**			
*bla*_CMY-2_	11 (15.1)	2 (4.5)	13 (11.1)
**Total**	25 (34.2)	6 (13.6)ˆ*	31 (26.5)

*^∗^*p* < 0.05 for gene detection rate of isolates from piglets vs. sows.*

### Molecular Typing

We used cluster analysis of the *bla*_CMY-2_-positive isolates to generate dendrograms from PFGE profiles (**Figure 1**). Six phylogenetic groups (designated A–F) each with more than 90% similarity were represented in these 13 isolates. Group C contained seven isolates, Group A two, and the other four groups one each.

Multilocus sequence typing analysis of the 13 *bla*_CMY-2_-positive isolates identified five different STs including a novel one (ST4234). The most prevalent STs were ST1121 (*n* = 7) and ST641 (*n* = 3). The remaining isolates were each of a single ST type (**Figure 1**). Interestingly, the seven ST1121 isolates from six sows and one piglet were all contained in Group C. The three ST641 isolates from sows were divided between two groups (**Figure 1**).

### Conjugation Assays and Plasmid Analysis

Eight *bla*_CMY-2_-positive transconjugants were successfully obtained from the seven ST1121 and one ST164 *E. coli* isolates. S1 nuclease-PFGE analysis identified a single plasmid in each of the seven transconjugants from ST1121 isolates. Two plasmids were identified in the transconjugant EC5422-25T derived from the ST164 isolate EC5422 (Supplementary Figure S1). Subsequently, Southern blot hybridization identified the *bla*_CMY-2_ gene located on a ∼290 kb plasmid in each of the transconjugants (Supplementary Figure S1). Replicon typing revealed the presence of the IncHI2 replicon in each of the eight transconjugants, but one of them (EC5422-25T) carried two replicons (IncHI2 and IncFII; **Figure 1**).

The IncHI2 plasmids of the ST1121 isolates shared indistinguishable RFLP profiles that were generated using *XbaI* digestion (data not shown). All of the transconjugants were resistant to FOX, CEF,CTX, AMP, KAN, and AMK. In addition, transfer of resistance to FFC, DOX, and SXT was also observed in the transconjugant EC5422-25T.

The metal susceptibility testing showed that the MICs of CuSO_4_ and AgNO_3_ for transconjugant EC5207-35T were higher than that of *E. coli* C600 (MIC_CuSO4_ = 12 vs. 8 mM; MIC_AgNO3_ = 1 vs. 0.0125 mM).

### Complete Sequence of Plasmid pEC5207

**FIGURE 2:**
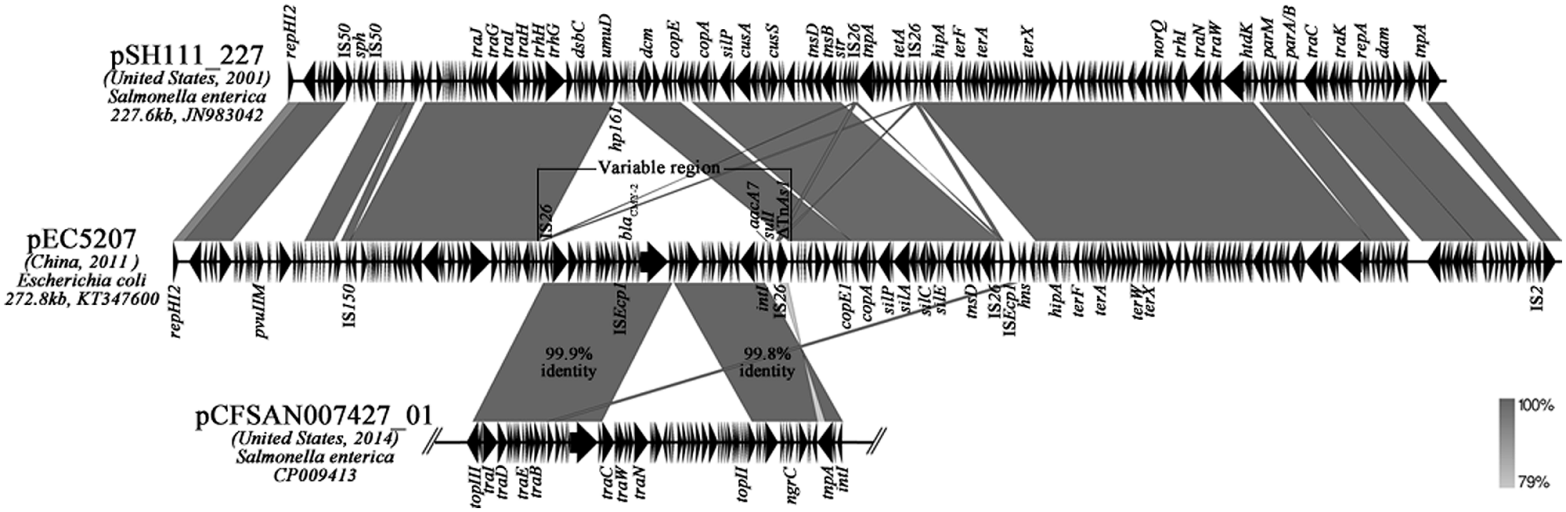
**Linear comparison of IncHI2 plasmids pEC5207, pSH111_227 and *bla*_CMY-2_-carrying IncA/C plasmid pCFSAN007427_01.** The arrows represent the positions and transcriptional direction of the ORFs. Regions of homology are shaded in gray.

We determined the DNA sequence of the plasmid derived from the *bla*_CMY-2_-positive transconjugants that were obtained from the seven ST1121 isolates. This plasmid pEC5207 is 272,865 bp in length with a GC content of 46.16% and harbors 253 predicted ORFs. The plasmid backbone is organized similarly to that of pSH111_227 (GenBank JN983042) from *Salmonella* sp. and encodes typical IncHI2 plasmid replication, partition, maintenance and transfer functions (**Figure 2**). The replication region of pEC5207 included a *repHI2* gene. The *parA* and *parB* genes were involved in the plasmid partition. Two *tra* transfer regions and a *hipAB* toxin-antitoxin gene cluster were associated with the transfer and maintenance function of pEC5207. Interestingly, pEC5207 also contained a large number of genes encoding resistance to heavy metals including tellurium (*terABCDEFWXYZ*), silver (*silABCEPRS*) and copper (*copABCE*; **Figure 2**).

The *bla*_CMY-2_ gene in pEC5207 is contained within a 48,888 bp variable region (**Figure 2**). The variable portion is located in a region between *umuD* and *dcm* in pEC5207, where is the *hp161* locus in pSH111_227. The variable region showed high homology (>99%) with two parts of the *bla*_CMY-2_-carrying IncA/C plasmid pCFSAN007427_01 (GenBank CP009413) from *Salmonella* and contained an IS*26* and a truncated Tn*As1* at the ends. In addition to *bla*_CMY-2_, a new class I integron harboring aminoglycoside resistance gene *aacA7* and sulfonamide resistance gene *sulI* was also found in this variable region.

## Discussion

Antimicrobial susceptibility testing showed different resistance levels to cephalosporins and other classes of antimicrobials in *E. coli* isolates from a farrowing farm in Southern China. The antimicrobial usage records for this farm showed that doxycycline and the first generation cephalosporin cefradine were frequently added to drinking water or compound feeds as prophylactics. Other antimicrobials such as ceftiofur, enrofloxacin, florfenicol, and sulfamethoxazole were also commonly used for treatment during production. This background may be favorable for developing resistance to cephalosporins and other antimicrobials in this farm. The ESC-resistant *E. coli* usually carry additional genes conferring resistance to other veterinary antimicrobial agents like quinolone, aminoglycoside and florfenicol. In our previous study, the ESBL and pAmpC genes were detected in 67.5% of the PMQR-positive *E. coli*, indicating a strong association between ESC and quinolone resistance (Liu et al., 2013a). The 16S rRNA methylase genes, especially *rmtB*, and the florfenicol resistance gene *floR* are also commonly identified in ESBLs and pAmpC-producing *E. coli* isolates (Kang et al., 2009; Wu et al., 2009; Yu et al., 2010; Deng et al., 2011; Liu et al., 2013b; Guo et al., 2014). The use of different antimicrobial agents may increase the potential risk for selection of multidrug resistant isloates, and contribute to the MDR phenotypes of *E. coli* isolates in this farm.

Compared with the isolates from piglets, the isolates from sows showed significantly higher resistance rates to FOX, CEF and CTX, as well as a relatively higher resistance rate to CAZ. This coincided with the higher occurrence of the *bla*_CMY-2_ and *bla*_CTX-M_ genes in the isolates from sows than in the isolates from piglets. Previous studies have shown that cephalosporin treatment can result in the selection of ESBL and AmpC producing *E. coli* in animals and aggravate the problem of cephalosporin resistance (Tragesser et al., 2006; Cavaco et al., 2008; Kanwar et al., 2013; Barton, 2014). The longer antibiotic exposure times of the sows may promote the persistence of ESBL/AmpC-producing *E. coli* in the gastrointestinal tract, thus resulting in the serious cephalosporin resistance and relatively high prevalence of ESBL/AmpC genes observed in sow isolates.

In this study the prevalence of the *bla*_CMY-2_ gene (11.1%) was lower than that of the *bla*_CTX-M_ genes (15.4%). However, this level was much higher than those in our previous study (2.9%; Fang et al., 2015) and others’ (1.0–3.0%) in China (Liu et al., 2007; Guo et al., 2014; Rao et al., 2014). Previous studies have shown that cefoxitin/ceftiofur-resistant isolates from *E. coli* and *Salmonella* had a high degree of association with the production of CMY enzymes (Barton, 2014). Therefore, the use of ceftiofur in this farm probably contributed to the selection of *bla*_CMY-2_-producing *E. coli* isolates. MLST typing revealed a major ST type, ST1121, in seven of the thirteen *bla*_CMY-2_-positive isolates. Since they shared a high similarity (>95%) in PFGE profiles, this suggested a clonal spread of these ST1121 isolates. Furthermore, plasmid analysis of the transconjugants from the seven ST1121 isolates revealed an indistinguishable IncHI2 plasmid harboring *bla*_CMY-2_ in these isolates. Thus, the clonal spread of ST1121 isolates harboring IncHI2 plasmid may play an important role in the dissemination of *bla*_CMY-2_ in this farm. Notably, among the 13 *bla*_CMY-2_-positive isolates, one of the ST1121 isolates and the ST2603 isolate were isolated from piglets for market, which would indicate that the *bla*_CMY-2_-positive isolates may have been introduced into other swine farms by piglet trading and therefore would accelerate the spread of *bla*_CMY-2_. Further surveillance is necessary to determine the prevalence of *bla*_CMY-2_-positive isolates in these swine farms.

Since its first identification on plasmid from *Klebsiella pneumoniae* (Bauernfeind et al., 1990), the *bla*_CMY-2_ gene has been associated with various plasmid types. In our previous surveillance study, the *bla*_CMY-2_ gene was identified on IncA/C, IncHI2, and IncX plasmids (Fang et al., 2015). In the present study, *bla*_CMY-2_-carrying IncHI2 plasmids were not only detected in the clonal ST1121 isolates but also in a ST164 isolate. Additionally, the strong association of *bla*_CMY-2_ with IncHI2 plasmid was also observed in *Salmonella* (Shi, 2015). This highlighted the role of the IncHI2 plasmid in the transfer of *bla*_CMY-2_. Besides *bla*_CMY-2_, IncHI2 plasmid is also associated with another pAmpC gene *bla*_CMY -8_ (Chen et al., 2007b). In addition, IncHI2 plasmids have been implicated in the spread of *bla*_CTX-M_ genes and are also frequently linked with the other antimicrobial resistance genes such as *bla*_SHV_, *bla*_IMP_, *bla*_VIM_, *armA, qnrA1, qnrS1*, and *qnrB2* (Garcia Fernandez et al., 2007; Veldman et al., 2010; Coelho et al., 2012). Together, our data indicate that the IncHI2 plasmid played a significant role in the dissemination of antimicrobial resistance. However, to the best of our knowledge, there was no complete sequence analysis of a *bla*_CMY-2_-carrying IncHI2 plasmid to date. Therefore, in order to further characterize the *bla*_CMY-2_-carrying IncHI2 plasmids of ST1121 isolates, plasmid pEC5207 was sequenced in our study.

This plasmid possesses a typical IncHI2 plasmid backbone organized similarly to that of pSH111_227. A portion of *hp161* in pSH111_227 was replaced with a *bla*_CMY-2_-harboring variable region in pEC5207. A sequence comparison revealed that this variable region might originate from IncA/C plasmid pCFSAN007427_01, and is flanked with IS*26* and a truncated Tn*As1*. Considering the potential for genetic rearrangements by insertion sequence and transposon (Hallet and Sherratt, 1997; Bennett, 2004), we speculate that IS*26* and Tn*As1* transposition into the *hp161* locus of a pSH111_227-like IncHI2 plasmid was followed by recombination with a pCFSAN007427_01-like *bla*_CMY-2_-carrying IncA/C plasmid. This could set the stage for the integration of *bla*_CMY-2_ into the IncHI2 plasmid.

Apart from the *bla*_CMY-2_ gene, pEC5207 also carried other resistance determinants for aminoglycosides (*aacA7*), sulfonamides (*sul1*), as well as heavy metals ions, such as Cu and Ag. And the result of susceptibility tests also confirm the functionality of pEC5207 in the transfer of copper and silver resistance. Copper is often present as trace element feed additives for animal production in China (Wang et al., 2013). Silver is used as disinfectants in water or surfaces in the animal production setting (Mourao et al., 2015). Under the selective pressure of heavy metals, cephalosporins, and other antimicrobials, plasmid pEC5207 could play a critical role in the persistence of host bacteria in the intestine of pigs, thereby increasing the risk for co-selection of isolates carrying *bla*_CMY-2_ gene. This may further explain the high prevalence of *bla*_CMY-2_ in this farm.

## Conclusion

The present study revealed a relatively high prevalence of *bla*_CMY-2_ in a farrowing farm in Southern China. The clonal spread of ST1121 type *E. coli* harboring an IncHI2 plasmid mediated the dissemination of *bla*_CMY-2_ in this farm. Nucleotide sequence analysis and comparisons indicated that the *bla*_CMY-2_-carrying IncHI2 plasmid pEC5207 may have been generated by recombination with an IncA/C plasmid. pEC5207 may play an important role in the persistence of host bacteria under the selective pressure of heavy metal and antimicrobials. Although limited to a farrowing farm, our study indicates that there is a serious risk of dissemination of the *bla*_CMY-2_ gene by clonal spread and piglet training. Broad and longitudinal studies to determine the prevalence of *bla*_CMY-2_-positive *E. coli* in swine farms are required in the future.

## Ethics Statement

This study protocol was reviewed and approved by the South China Agriculture University Animal Ethics Committee. The owner of the farm from which rectal swabs were taken gave permission for their animals to be used in this study.

## Conflict of Interest Statement

The authors declare that the research was conducted in the absence of any commercial or financial relationships that could be construed as a potential conflict of interest.

## Footnotes

1 http://www.lahey.org/studies/webt.asp

2 http://mlst.ucc.ie/mlst/dbs/Ecoli

3 http://blast.ncbi.nlm.nih.gov
