# Supplementary material for: Prevalence of extended-spectrum cephalosporin-resistant Escherichia coli in a farrowing farm: ST1121 clone harboring IncHI2 plasmid contributes to the dissemination of blaCMY-2
Source: Front Microbiol. 2015 Nov 3;6:1210. doi: 10.3389/fmicb.2015.01210 (PMC4630580; doi:10.3389/fmicb.2015.01210)
Supplement: Supplementary file 1 [file Image_1.PDF]

Supplementary Material

Prevalence of extended-spectrum cephalosporinase (ESC)-producing *Escherichia coli* in a farrowing farm: ST1121 clone harboring IncHI2 plasmid contribute to the dissemination of *bla*$_{CMY-2}$

Hui Deng, Hong-bin Si, Jian Sun, Liang-Xing Fang, Run-shi Yang, Ya-Hong Liu, Xiao-Ping Liao*

*Corresponding author: Xiao-Ping Liao, E-mail: xpliao@scau.edu.cn

Supplementary Figure

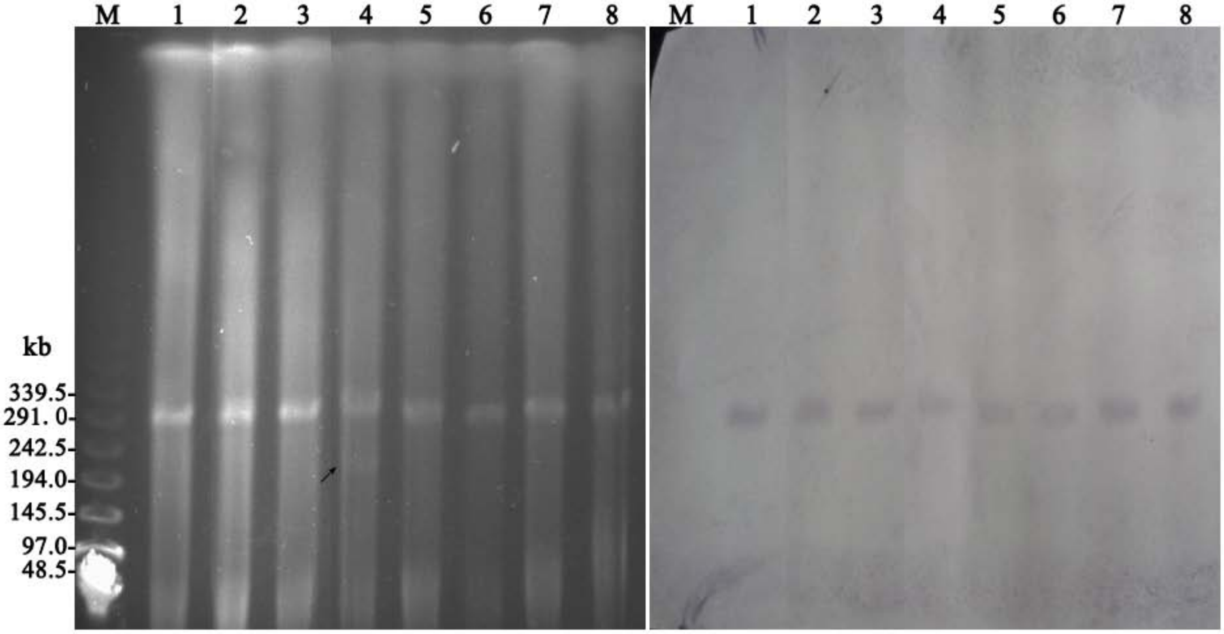

Figure S1. The size of *bla*$_{CMY-2}$-carrying plasmids in the eight transconjugants. Left panel: S1 nuclease-PFGE of the *bla*$_{CMY-2}$-positive transconjugants. Right panel: subsequent Southern blot hybridization with *bla*$_{CMY-2}$-specific probe. Lanes: M, molecular size markers (Lambda Ladder PFG marker; New England Biolabs);1, EC5207-35T; 2, EC3602-166T; 3, EC5417-21T; 4, EC5422-25T; 5, EC5423-24T; 6, EC6419-50T; 7, EC5320-29T; 8, EC1125-138T.
